# Construction of a high-resolution genetic map and identification of single nucleotide polymorphism markers relevant to flower stalk height in onion

**DOI:** 10.3389/fpls.2023.1100691

**Published:** 2023-01-31

**Authors:** Yanwei Li, Yumeng Huo, Yanyan Yang, Zhenbao Wang, Yaling Sun, Bingjiang Liu, Xiong Wu

**Affiliations:** Key Laboratory for Biology of Greenhouse Vegetables of Shandong Province/National Center for Vegetable Improvement (Shandong Branch), Vegetable Research Institute, Shandong Academy of Agricultural Sciences, Jinan, China

**Keywords:** double haploid (DH), SLAF sequencing, genetic map, QTL analysis, onion

## Abstract

**Introduction:**

Onion (Allium cepa L., 2n=16) is an economically and nutritionally important vegetable crop worldwide. Construction of a high-resolution genetic map and map-based gene mining in onion have lagged behind other vegetable crops such as tomato and pepper.

**Methods:**

In this study, we constructed a high-resolution genetic map of onion using 321 F2 individuals from a cross between two double haploid lines DH-1×DH-17 and employing specific length amplified fragment (SLAF)-seq technology. The genetic map containing 10,584 polymorphic SLAFs with 21,250 single nucleotide polymorphism (SNP) markers and 8 linkage groups was developed for onion, which spanned 928.32 cM, with an average distance of 0.09 cM between adjacent markers.

**Results:**

Using this map, we carried out QTL mapping of Ms locus related to the male-fertile trait and reproduced previous mapping results, which proved that this map was of good quality. Then, four QTLs (located on LG2, LG5, and LG8) were detected for flower stalk height, explaining 26.60% of the phenotypic variance. Among them, we proposed that 20 SLAF markers (in three QTLs) of flower stalk height trait were effective favorable allelic variant markers associated with heterosis.

**Discussion:**

Overall, the genetic map was structured using SLAF-seq based on DH lines, and it is the highest-quality and highest-resolution linkage map of onion to date. It lays a foundation for the fine mapping and candidate gene identification of flower stalk height, and provides new insights into the developmental genetic mechanisms in onion breeding.

## Introduction

Onion (*Allium cepa* L.) is a diploid species (2n=16) that belongs to the genus *Allium*, family Amaryllidaceae, and it is an economically and nutritionally important vegetable crop worldwide (Khosa et al., 2016). Onion is widely distributed, grown throughout the North and South, and is one of the main domestic vegetables, but also one of the main export vegetable crops in China. According to the statistics of the Food and Agriculture Organization (FAO, 2020), China has the world’s largest onion production area and yield, accounting for approximately 30% of the world’s total area and yield. Onion is a biennial plant, and decreasing the long breeding cycle is desirable for efficient onion breeding. Therefore, many studies focus on the molecular markers of fertility identification related to actual breeding (King et al., 1998; Park et al., 2013).

Our team previously developed molecular markers that are co-segregating with the male sterility gene and restorer gene, such as cDNA marker WHR240 (Huo et al., 2012), SCAR markers DNF-566 and DNS-357 (Yang et al., 2013), and multiple PCR marker AcSKP1 (Huo et al., 2015). These markers have been verified in open-pollinated (OP) populations with different genetic backgrounds, and can be directly used to identify the genotype of nuclear male sterility locus in onion. However, research on genetic linkage mapping and markers in the onion requires a great deal of time, due to its huge genome size (approximately 15 Gb), large number of repetitive sequences, and high heterozygous genetic background (Labani and Elkington, 1987; King et al., 1998; Jakse et al., 2005; Finkers et al., 2021).

To date, progress on onion genomic and genetic maps has been reported as follows (King et al., 1998; Martin et al., 2005; Duangjit et al., 2013; Jo et al., 2017; Choi et al., 2020; Cho et al., 2021; Finkers et al., 2021). The first onion genetic map was constructed by King et al. (1998) using dominant markers [randomly amplified polymorphic DNA (RAPD) and amplified fragment length polymorphism (AFLP) markers], which consisted of 116 markers, distributed in 12 linkage groups with an average genetic distance of 9 cM. Subsequently, Martin et al. (2005) developed 100 new molecular markers based on the rice expressed sequence tags library with high similarity and integrated them into the onion genetic map reported above using the chromatid replacement line of scallion, constructing a genetic map of 14 linkage groups with a total length of 1,907 cM. Duangjit et al. (2013) constructed a genetic map covering 936 single nucleotide polymorphism (SNP) markers and 10 linkage groups by library analysis on the Roche 454 platform of the leaves, bulbs, roots, and flower buds from two inbred lines. Jo et al. (2017) developed 202 SNP markers using an F_2_ population by genotyping by sequencing (GBS) to construct a genetic map with a genetic distance of 1,383 cM and an average marker distance of 8.08 cM. Choi et al. (2020) performed GBS and high-resolution melting (HRM) analyses on F_2_ onion plants and constructed an onion genetic linkage map with 319 SNPs and 34 HRM markers, consisting of 8 linkage groups and covering 881.4 cM with an average marker interval of 2.5 cM. Through quantitative trait loci (QTL) analysis, a major QTL, *qAS7.1*, for onion anthocyanin synthesis and two significant QTLs, *qAC4.1* and *qAC4.2*, for anthocyanin content were identified. Subsequently, Cho et al. (2021) developed 652 molecular markers using the above same F_2_ population for constructing genetic maps with increased resolution. Fujito et al. (2021) constructed a high-density linkage map in *Allium cepa* using 1,435 unigene markers obtained from the transcriptome information of a 96 F_2_ plants (from a cross between the A. *cepa* DH lines). The first *de novo* genome sequence (14.9 Gb with an N50 of 464 Kb) of onion was assembled based on Illumina short read sequencing by Finkers et al. (2021), and covered approx. 91% of the expected genome size with 72.4% of the genome identified as repetitive sequences and 20% of the putative (retro) transposons. Of this, 2.4 Gb was ordered into eight linkage groups, which was a valuable resource for research and breeding. However, fine mapping of onion traits is difficult due to the low densities of the genetic markers.

Advances in genotyping-by-sequencing have provided an excellent opportunity to increase the resolution of QTL mapping by increasing the numbers of markers in recent years (Varshney et al., 2009). Specific length amplified fragment (SLAF) sequencing (SLAF-seq), a high-resolution strategy of large-scale *de novo* SNP discovery and genotyping, was first described by Sun et al. (2013). SLAF-seq has been proved to be an effective method for genetic map construction and has wide applications in cucumber (Wei et al., 2014; Zhu et al., 2016), cotton (Zhang et al., 2016; Ali et al., 2018), peanut (Hu et al., 2018), pea (Zheng et al., 2018), soybean (Zhang et al., 2020; Mendoza et al., 2021), and eggplant (Wei et al., 2020). Compared with GBS, SLAF-seq has the following advantages: I. SLAF library was prepared by the double enzyme protocol and double barcode system, while GBS by the single enzyme. The DNA fragments sheared by double enzyme digestion were more uniformly distributed in the genome and the flexible enzymes combinations made it easier to control the number of markers; II. the marker density of SLAF was of 50-80/Mb, while of GBS was 5-40/Mb (Elshire et al., 2011; Sun et al., 2013). Therefore, SLAF-seq technology could provide a high-resolution strategy for SNP discovery, which is more suitable for widespread use in large-scale genotyping study. Considering the number of markers, the quality of markers, number of samples (321 samples) and research funding (the huge and complex genome of onion), SLAF-seq is the optimal choice for large-scale molecular marker development and high-resolution linkage map construction, especially in onion for which no reference genome information is available.

In this study, we constructed an F_2_ population from a cross between two double haploid lines, DH-1 and DH-17. Using the F_2_ population containing 321 individuals and SLAF-seq technology, we constructed a high-resolution genetic map in onion with 21,250 SNP markers. To verify the quality of the map, we performed QTL mapping of a male fertile trait. Then, we identified the QTLs related to flower stalk height (FSH) trait and explored their QTL effects to reveal the genetic mechanism of the FSH of onion, initially explaining its heterosis mechanism.

## Materials and methods

### Plant materials and phenotypic evaluation

**Figure 1:**
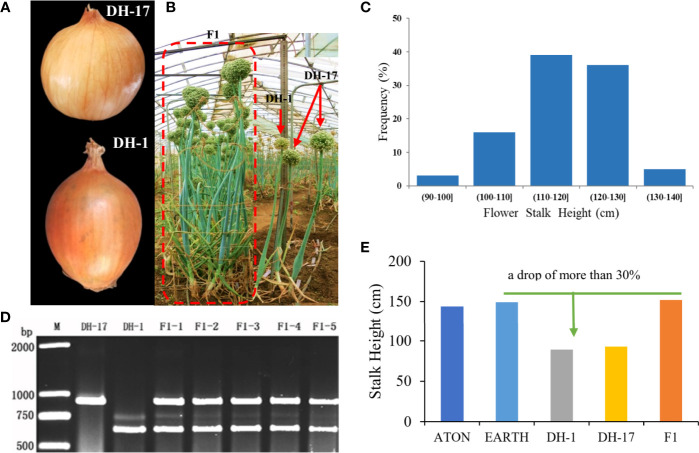
Phenotypes of the parental inbred lines and the F_1_, F_2_ individuals. **(A)** Paternal line DH-17 and maternal line DH-1; **(B)** Plant phenotypes of both parents and F_1_ individuals; **(C)** Phenotypes evaluation of flower stalk height for the F_2_ individuals; **(D)** Genotypes of both parents and partial F_1_ individuals by the PCR markers DNF-566 and DNS-357; **(E)** Height of flower stalk for the original hybrids, DH lines and F_1_.

Two onion double haploid (DH) lines were used in this study as parents. DH-1, whose male-sterility locus was S (*msms*) and whose phenotype was sterile, was induced from the Japanese hybrid ATON (Takii Seed, **Figure 1A**) *via in vitro* gynogenesis (Liu et al., 2012). Through flow cytometry analysis of leaf tissue, we preliminarily identified DH lines DH-1 and DH-17. To ensure that the DH lines were naturally doubled and no other sources such as somatic cells, we also performed PCR detection with *Ms*/*ms* co-segregated markers DNF-566 (F: 5´-TACAGATTTGTTTATCTTCTTCTTCTTCT-3´; R: 5´-TTCATTTGTTAGGATGTACTCTTACC-3´) and DNS-357 (F: 5´-TCAGTATCAATAGAAGGAATCAC-3´; R: 5´-GTATACCATTGGTACTTGATGCA-3´, Yang et al., 2013). DH-17, which was for S (*MsMs*) with a fertile phenotype, was derived from the Japanese hybrid EARTH (Takii Seed, **Figure 1A**). F_1_ hybrid came from a crossed between DH-1 (female parent) and DH-17 (male parent) were grown in greenhouses in early September of 2015; then a total of 321 F_2_ individuals was derived from a self-crossing of F_1_ were randomly selected to construct the genetic map. The parents and 321 F_2_ individuals were grown in greenhouses in early September of 2017 at the Shandong Academy of Agricultural Sciences, Jinan, China. The genotype was identified for each individual by DNF-566 and DNS-357 markers (Yang et al., 2013), and individuals were identified as male sterile or fertile through inspection of pollen at mature anthers stage in June of 2019. The phenotypic data for FSH was collected in June of 2019. The FSH is defined as the height from the ground surface to the top of the stem, excluding the inflorescence.

### DNA extraction

Young leaves from the 321 F_2_ individuals and two parents were collected, and then stored in a -80°C freezer. The total genomic DNA was extracted from each sample according to the cetyltrimethyl ammonium bromide (CTAB) method (Murray and Thompson, 1980). The concentration and quality of total DNA were evaluated by electrophoresis on 1% agarose gels and an ND-1000 spectrophotometer (NanoDrop, Wilmington, DE, USA).

### SLAF library construction and sequencing

SLAF-seq with some improvements was applied to genotype onion 321 F_2_ individuals and two parents in 2018 (Sun et al., 2013). In the absence of reference genome, the dendrobium (https://www.ncbi.nlm.nih.gov/assembly/GCA_001605985.2), a closely related species, was finally selected as the reference genome for electronic enzymatic digestion prediction according to the onion genome size and GC content information. *Hae*III and *Eco*RV-HF (New England Biolabs, NEB, USA) were selected as endonucleases to digest the genomic DNA. A single nucleotide (A) overhang was added to the digested fragments using Klenow Fragment (3´→5´, exo-) and dATP at 37°C. Then, duplex tag-labeled sequencing adapters were ligated to the A-tailed fragments using T4 DNA ligase. PCR reactions were run using diluted restriction-ligation DNA, dNTPs, Q5^®^ high-fidelity DNA polymerase, and universal PCR primers (F: 5´-AATGATACGGCGACCACCGA-3´, R: 5´-CAAGCAGAAGACGGCATACG-3´; PAGE-purified, Life Technologies). PCR products were purified using Agencourt AMPure XP beads (Beckman Coulter, High Wycombe, UK), and pooled samples were separated by 2% agarose gel electrophoresis. The digested fragments ranging from 414-444 base pairs (with indexes and adaptors) in size were excised and purified using a QIAquick gel extraction kit (Qiagen), and then, pair-end sequencing (100 bp) of the gel-purified products was performed on an Illumina HiSeq 2500 system (Illumina, Inc., San Diego, CA, USA) according to the manufacturer’s recommendations.

### SLAF-seq data analysis and genotyping

SLAF-seq data were assessed by the software developed by Sun et al. (2013), and SLAF marker identification and genotyping were performed according to procedures described by Sun et al. (2013). Firstly, after filtering out low-quality reads (quality score < 20e), raw reads were sorted to each progeny according to duplex barcode sequences; then trimming the barcodes and the terminal 5-bp positions from each high-quality reads, clean reads were clustered by similarity above 90%. Sequences clustered together were defined as one SLAF locus (Zhang et al., 2015). Secondly, single nucleotide polymorphism (SNP) loci of each SLAF locus were detected between parents. SLAFs with more than 5 SNPs were filtered out, and SNP loci of each SLAF locus were detected between parents by using the GATK software kit (McKenna et al., 2010). Alleles of each SLAF locus were then defined according to parental reads with sequence depth >319.07-fold, while for each progeny the reads with sequence depth >29.12-fold were used to define alleles. For diploid species, one SLAF locus can contain at most 4 genotypes, so SLAF loci with more than 4 alleles were defined as repetitive SLAFs and discarded. Only SLAFs with 2 to 4 alleles were identified as polymorphic and considered potential markers. All polymorphism SLAFs loci were genotyped with consistency in the parental and progeny SNP loci. Because the mapping populations were derived from two onion DH parents with a genotype of aa or bb, only the polymorphic SLAFs were analyzed on the basis of the F_2_ population type (aa × bb).

Genotype scoring was performed by a Bayesian approach to ensure the genotyping quality (Sun et al., 2013). *A posteriori* conditional probability was calculated using the coverage of each allele and the number of SNP, and genotyping quality score translated from the probability was used to select qualified markers. Then, low-quality markers were counted and the worse marker or individual were deleted. High-quality SLAF markers for the genetic mapping were filtered by the following criteria: (1) contained more than 5 SNPs; (2) markers with more than 70% missing data, (i.e., complete degree of markers in each progeny below 70% discarded); (3) markers with significant separation distortion (*p* > 0.05) by using chi-square test; (4) parents’ sequence depth of less than 10×. The remaining high-quality SLAFs were subsequently used for constructing the linkage map.

### Linkage map construction

To ensure efficient construction of the high-resolution and high-quality map, the SLAF markers were correctly ordered by the HighMap strategy, and genotyping errors were corrected by the SMOOTH algorithm (van Os et al., 2005; Liu et al., 2014). First, the recombination rate and maximized logarithm of odds (MLOD) value between two points were calculated. Then, the interval of the MLOD value was determined by arranging the MLOD values of those markers from the smallest to largest, and the highest MLOD values among the markers were in the same linkage group (LG). Finally, all SLAF markers (MLOD > 5) were divided into 8 onion linkage groups (LGs; each linkage group was considered as a chromosome) to construct the genetic map (Zhang et al., 2015). Taking LG as a unit, the regression algorithm/minimum spanning tree (MST) algorithm was selected to estimate the genetic distance between adjacent markers, and the optimal linear order of markers in LGs was obtained by regression sequencing analysis (Liu et al., 2014). Then, the k-nearest neighbor algorithm was applied to impute missing genotypes (Huang et al., 2011), and the Kosambi mapping function was used for estimating map distances (cM) (Kosambi, 1943).

### QTL mapping

The composite internal mapping (CIM) analysis within the R/QTL software was applied for QTL mapping of plant male fertility (Broman and Sen, 2009). The logarithm of odds (LOD) threshold was determined using the 1000 permutations test (PT, *P*<0.05) to evaluate the statistical significance of each QTL. To ensure that major and minor effect QTLs could be identified, different LOD scores were adopted. First, an LOD threshold corresponding to 0.99 confidence was considered, followed by 0.95 and 0.90. Then, if no QTL interval was detected, the PT result was not considered, and the threshold was manually lowered to 3.0, 2.5, and 2.0 successively. QTLs were normally named according to their linkage group locations and trait names.

## Results

### SLAF-seq data and SLAF marker analysis

SLAF sequencing of the 321 F_2_ population, including the two parents, generated a total of 857.58 GB clean data comprising 4.29 G reads of approximately 100-bp paired-end sequencing (**Table 1**). Among these reads, 95.70% achieved or exceeded a quality score of 30 (Q30, indicating a 0.1% chance of an error and 99.9% confidence), and the guanine-cytosine (GC) content was 40.54%. There were 131,741,300 reads from male parent DH-17 and 119,231,892 reads from female parent DH-1, and the average number of reads from the F_2_ population was 11,567,116. Specifically, the reads of rice (*Oryza sativa* L. var. Japonica, genome size ≈ 382 Mb), which were used as a control to estimate the validity of library construction, were 6,852,482 with 95.46% Q30 bases and 45.60% GC (**Table 1**).

**Table 1:** Summary of the SLAF-sequencing data.

Sample ID	Total Reads (Mb)	Total Bases (Gb)	Q30 (%)	GC (%)
DH-17	131.74	26.35	95.56	41.41
DH-1	119.23	23.85	95.86	39.88
F_2_-progeny	11.57	2.31	95.70	40.53
Total	4,287.90	857.58	95.70	40.54

**Figure 2:**
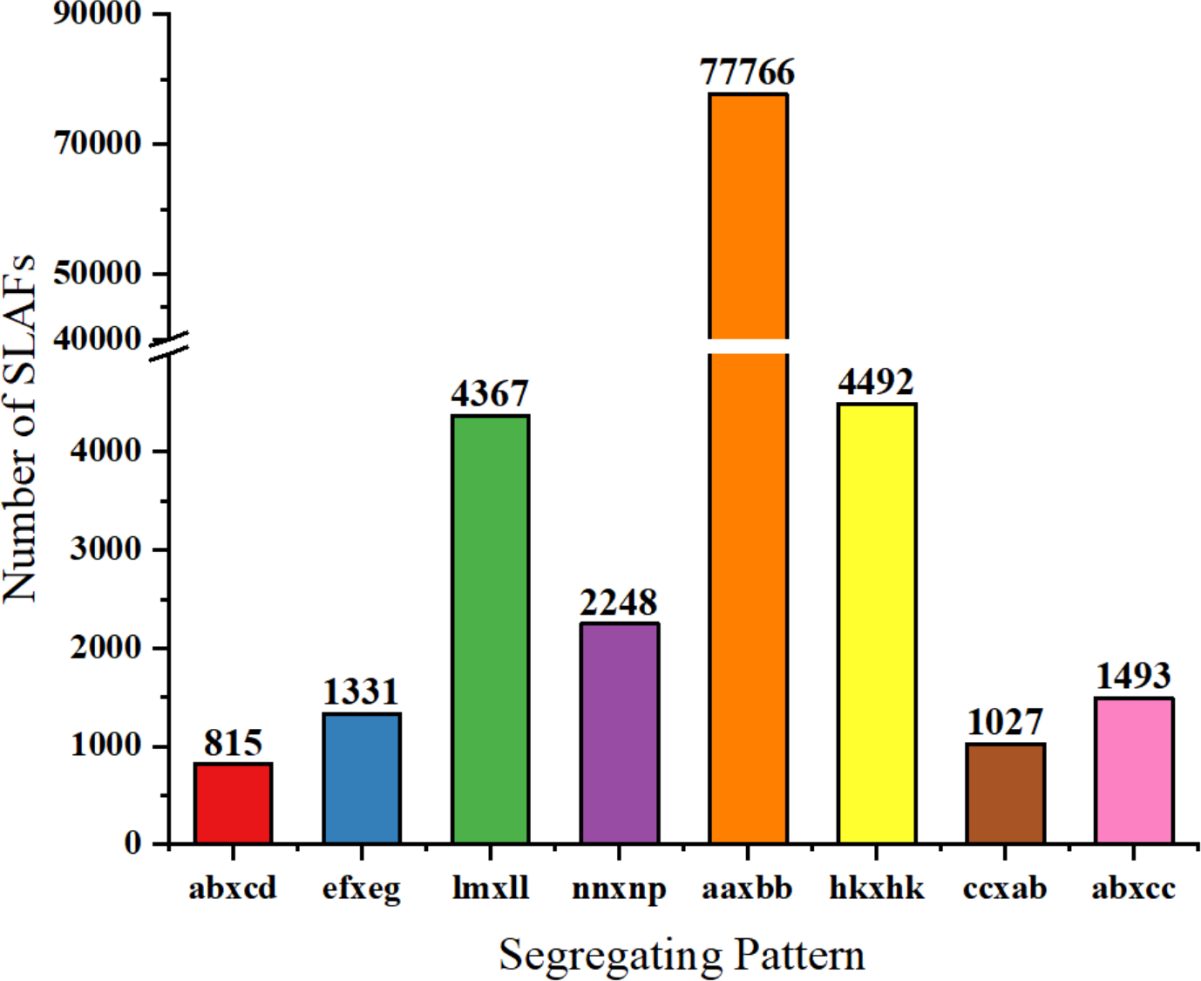
Genotype distribution of SLAF markers in onion. The Y-axis indicates the number of SLAF markers, x-axis indicates the segregation patterns of markers.

The number of SLAFs (based on the SLAF development process, with the reads at the same location being defined as a SLAF tag) in the male, female parent, and F_2_ population were 533,049, 458,885, and 323,656, respectively. The average sequencing depths of the male, the female parent, and F_2_ were 90.84×, 100.19×, and 13.68×, respectively. Among the 692,676 SLAFs developed, 155,067 were polymorphic SLAFs with a rate of 22.39% (**Supplementary Table S1**). Of those 155,067 polymorphic SLAF tags, 93,539 were successfully encoded and grouped into eight segregation patterns (ab×cd, ef×eg, ab×cc, cc×ab, hk×hk, lm×ll, nn×np and aa×bb; **Figure 2**; **Supplementary Table S2**). Due to the F_2_ population obtained by selfing the F_1_ of a cross between two parents with the homozygous genotype of aa or bb, only the F_2_ plants with the aa×bb segregation pattern were valid polymorphic SLAFs. There were 77,766 SLAFs accounting for 83.14% of the total encoded markers that were categorized with a segregation pattern of aa×bb in this study (**Figure 2**).

### A high-resolution genetic linkage map

To produce a high-quality map, low-quality SLAFs with a parental sequencing depth < 10×, SNP number > 5, segregation distortion *p* < 0.05, and MOLD between neighboring markers < 5 were discarded. To construct a genetic map, 10,584 high-quality SLAFs including 21,250 SNPs were retained (**Table 2**). For the map, the total distance of the genetic map was 928.32 cM, and the average interval between markers was 0.09 cM (**Table 2**). The average depths of the SNPs for female, male parent, and F_2_ population were 313.74×, 324.40×, and 29.12×, respectively.

**Table 2:** Basic information for high-resolution genetic map of onion.

LG ID	SLAF Num.	SNP Num.	Total Dis. (cM)	Average Dis. (cM)	Gaps<=5	Max Gap (cM)
LG1	1,185	2,336	115.29	0.10	100%	3.60
LG2	1,704	3,396	131.33	0.08	99.94%	6.27
LG3	1,689	3,369	140.43	0.08	100%	2.18
LG4	1,425	2,922	102.40	0.07	100%	2.92
LG5	1,209	2,368	100.99	0.08	100%	1.35
LG6	198	352	103.59	0.53	100%	2.59
LG7	1,500	3,059	96.24	0.06	100%	2.12
LG8	1,674	3,448	138.05	0.08	100%	2.27

**Figure 3:**
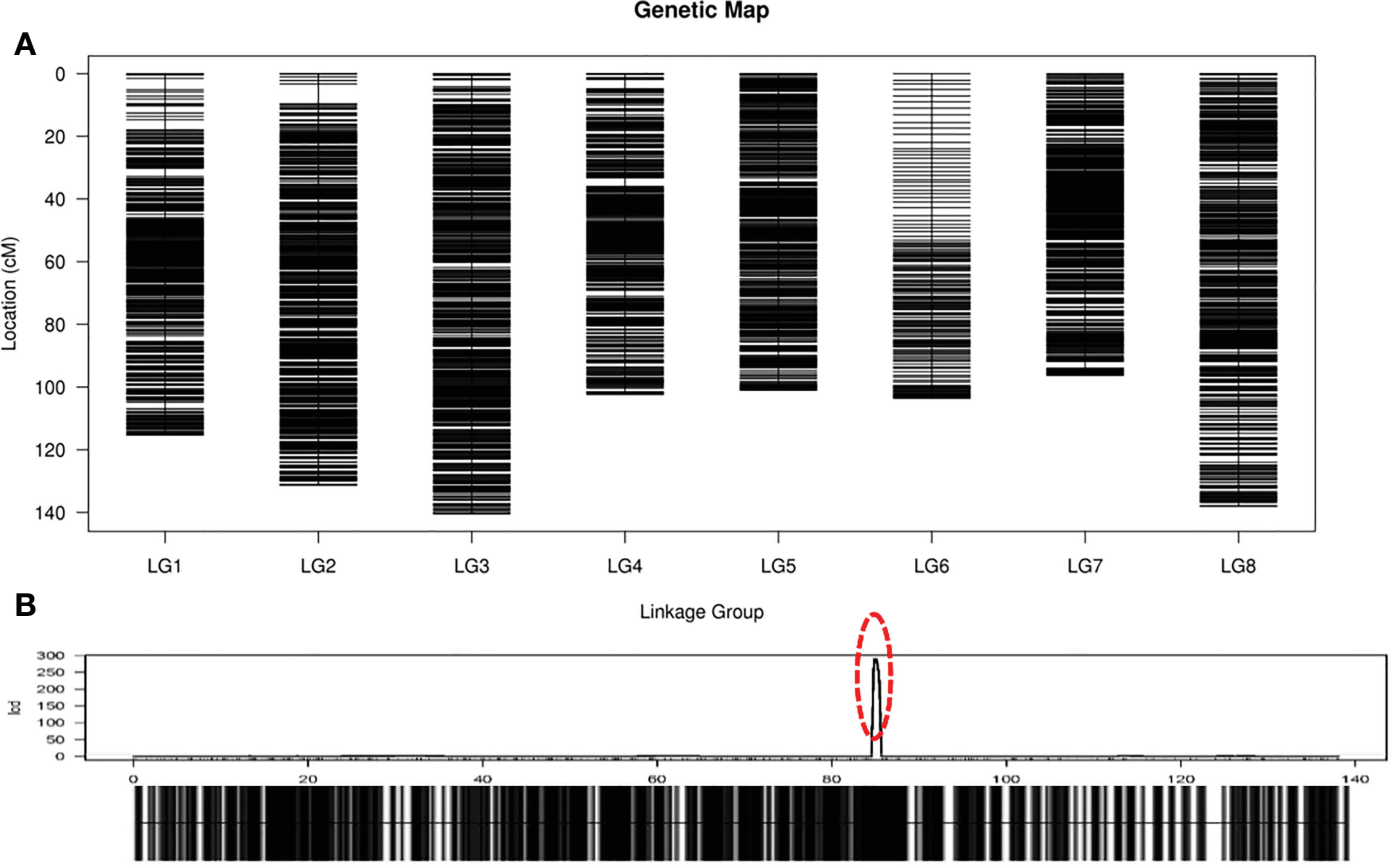
High-resolution genetic map of onion. **(A)** Genetic linkage map of onion. The black bars on each linkage group represent SLAF markers on the map; **(B)** Locations of male-fertile trait on genetic map. The red circle indicates the site of male-fertile trait.

Based on those analyses, we constructed a new high-resolution onion genetic map containing 8 linkage groups (LGs, **Figure 3A**). The linear arrangements and the genetic distances of markers in each LG were analyzed using the HighMap software. The average integrity of the mapped markers was 96.01%, which adequately ensured the accuracy of the genotyping. The genetic distances in the map spanned 96.24 cM (LG7) to 140.43 cM (LG3), with the number of SLAF markers in each LG ranging from 198 (LG6) to 1,704 (LG2) and SNP marker numbers ranging from 352 (LG6) to 3,448 (LG8, **Table 2**). The average number of SLAF and SNP markers in all LGs were 1,323 and 2,656, respectively, and markers spanned an average length of 116.04 cM. Satisfactory uniformity in the distribution was obtained, with gaps < 5 cM constituting 99.94% of the total LGs, and the average max gap was 2.91 cM (**Table 2**).

### Male-fertile phenotypic characterization of the genetic population

Performing PCR to detect the DH lines genotype with *Ms*/*ms* co-segregated markers DNF-566 and DNS-357 (Yang et al., 2013), the results showed that the *Ms* locus of DH-1 was S *(msms)*, which was a sterile phenotype; the genotype of DH-17 was S (*MsMs*) with a fertile phenotype (**Figure 1D**). According to plant genotype, the F_2_ population was divided into the following three groups (**Supplementary Excel S1**): *MsMs* of 75 individuals, *Msms* of 166 individuals, and *msms* of 80 individuals, with a 1:2:1 segregation ratio. By chi-square test, the separation ratio of fertile (241 individuals) and sterile plants (81 individuals) was 3:1. These results suggest that the onion *Ms*/*ms* trait is controlled by one dominant loci, which was consistent with previous conclusions (Jones and Clarke, 1943).

### QTL mapping reproducibility in the F_2_ population

**Figure 4:**
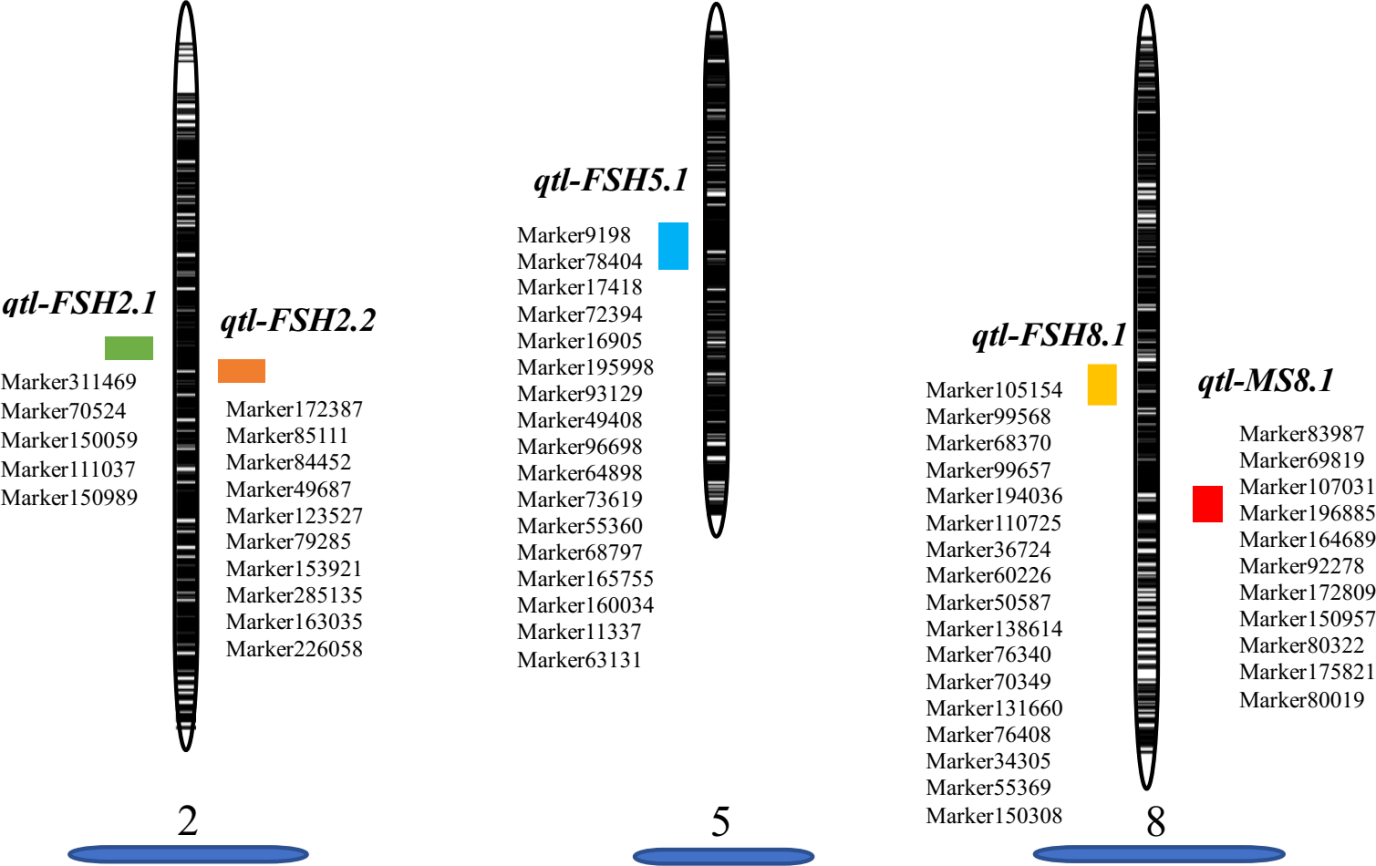
Graphic view of the distribution of markers associated with related traits on onion LGs 2, 5, 8.

Before conducting QTL mapping for onion agronomic traits on the basis of the high-resolution genetic map, we performed QTL mapping of a male fertile trait to verify the quality of the map. Only one QTL locus for male-fertile trait, designated *MS8.1*, was mapped on LG8 (**Figure 3B**), which could explain 110.31% of the observed phenotypic variation (PVE). The genetic distance interval of *qtl-MS8.1* ranged from 84.83 to 85.42 cM, and 11 SLAF markers were uncovered within this locus. The additive and dominant effects for *qtl-MS8.1* were -0.49 and 0.49, respectively, and the LOD threshold value of the QTL was 241.64. Based on the study of the DNF-566 and RNS-357 markers by Yang et al. (2013), (Kim et al., 2014; Kim et al., 2015) developed one co-dominant marker jnurf13 [an insertion deletion (indel) marker] in 2014 and proposed in 2015 that the bulb onion DNA mismatch repair (*AcPMS1*) gene was the most probable candidate for regulating fertility restoration. Huo et al. (2015) identified the putative S-phase kinase-associated protein 1 (SKP1) protein in linkage disequilibrium with *Ms* locus. In conclusion, it is likely that the male-fertility QTL locus located in this study is the same as that mapped by King et al. (1998). The SLAF marker distribution results in this study are presented in **Figure 4**. Detailed sequence information and the positions of SNPs uncovered within SLAFs are presented in **Supplementary Table S3**.

### QTLs mapping of the FSH trait

**Figure 5:**
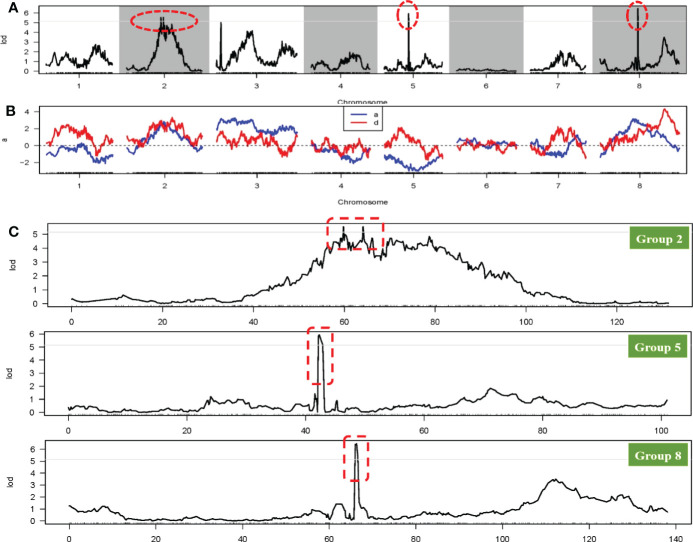
QTL locations of flower stalk height trait using the genetic map. **(A)** QTL analysis of the flower stalk height trait on eight linkage groups; **(B)** Phenotypic contribution rate for variation of the flower stalk height trait; **(C)** LOD scores for the QTLs of the flower stalk height trait on the 2^th^, 5^th^ and 8^th^ LG. The red circle or box all indicate the QTL regions related to flower stalk height trait.

The FSH phenotypic data for all onion materials (including two original induced hybrids, two parents, and F_1_ and F_2_ populations) are presented in **Figures 1B, C**. The FSH of double haploids DH-1 and DH-17 was lower than FSH of the original inducing hybrids from which the parental DH lines were developed by more than 30%. The FSH of F_1_ plants from cross with DH-1×DH-17 was restored to FSH of the original inducing hybrids, and the height of the F_1_ plants were highly uniform and consistent (**Figure 1E**). The height of the flower stalks in this study were measured using a ruler. FSH ranged in a spectrum from short stalk to tall stalk in the segregating populations and was thus assessed on a 1-5 height scale. There were a tiny percentage of F_2_ individuals within the range of 90.01-100 cm (3%, 11 individuals) and 130.01-140 cm (5%, 17 individuals), then 16% in the range of 100.01-110 cm (51 individuals), and the majority percentage within the range of 110.01-120 cm (39%, 125 individuals) and 120.01-130 cm (36%, 117 individuals). Analyses of the FSH data showed a normal distribution in the F_2_ population (**Figure 1C**; **Supplementary Excel S1**). According to the genotype of the onion F_2_ population from the SLAF-seq data, 49 SLAF markers (located in 4 QTLs) were detected and considered as favorable allelic variant (FAV) markers for flower stalk height. The LGs, starting positions, ending positions, SLAF markers, LODs, additive values, dominance values, and PVEs are shown in **Figures 4**, **5**, **Table 3** and **Supplementary Table S4**.

**Table 3:** Analysis of quantitative trait loci (QTL) for flower stalk height in onion.

QTL	LG ID	Linkage Map Pos. (cM)	Interval Size (cM)	LOD	Max LOD	SLAF Num.	ADD	DOM	PVE%
*qtl-FSH2.1*	LG2	59.82-59.83	0	5.15	5.55	5	2.80	2.98	7.19
*qtl-FSH2.2*	LG2	64.11-64.27	0.15	5.15	5.55	10	2.86	2.48	6.65
*qtl-FSH5.1*	LG5	42.16-42.90	0.74	5.15	5.94	17	-2.99	0.83	6.45
*qtl-FSH8.1*	LG8	66.01-66.60	0.59	5.15	6.48	17	3.00	1.60	6.28
Total	–	–	–	–		49		7.89	26.57

LOD, logarithm of odds; ADD, additive; DOM, dominance; PVE, phenotypic variance explained.

**Figure 6:**
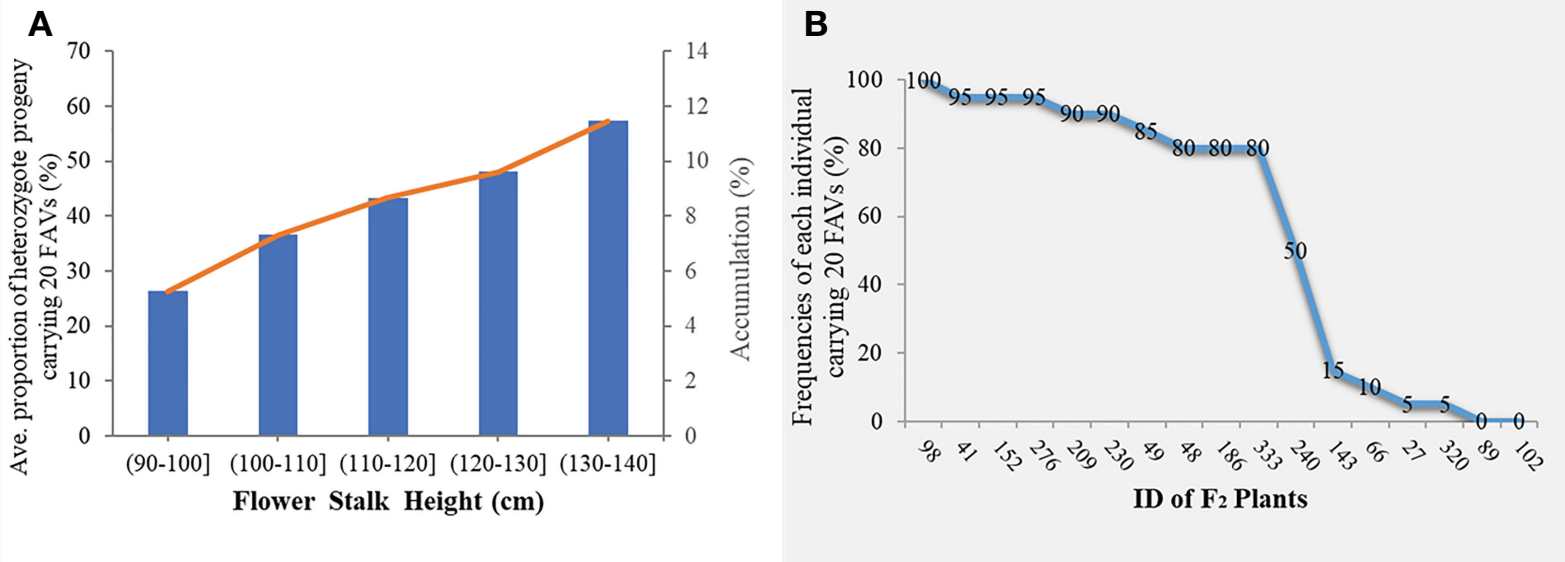
Statistical analysis of effective favorable allele varieties (FAVs) in the F_2_ population. **(A)** Average (accumulation) proportion of heterozygote progeny carrying effective FAV markers in the F_2_ population. The orange curve indicates the trend curve between cumulative proportion and flower stalk height; **(B)** Frequencies of the 20 FAV loci in the F_2_ individuals with the height range from 130-140 cm.

To avoid false-positive sites caused by repetitive sequences or splicing errors, and further narrow down the range of FAV markers, in our opinion, those SLAF markers that were beneficial to the flower stalk height of heterozygous progeny could be considered as effective FAV markers and should be preferentially selected for analysis. Since there was no significant difference in FSH between the parents used to construct the population, we only calculated the proportion of heterozygote progeny carrying FAV markers for the perspective of heterosis effect. The results showed that the proportion of heterozygous individuals carrying 20 markers were positively correlated with the height of flower stalk in F_2_ population were selected from the all 49 SLAF markers (**Supplementary Table S5**). After evaluating the proportion of F_2_ heterozygous individuals that carried the FAV marker by two measures, average proportion and total proportion in F_2_ population, we proposed that there were 20 SLAF markers (in 3 QTLs) that could be considered as effective FAV markers of FSH (**Figure 6A**; **Table 4**). Those 3 QTLs related to the FSH trait were distributed on linkage groups LG2 and LG8 (**Figures 4**, **5**; **Table 3**). The genetic distance interval of the QTLs ranged from 0 to 0.59 cM. The PVE by the 3 QTLs ranged from 5.17-7.19%, and the accumulated PVE was 19.90%. The LOD value of the 20 SLAF markers ranged from 5.17-6.84 (**Table 5**). The number of SNP loci within each SLAF varied from 1 to 5. Detailed sequence information and alignment positions for all markers are presented in **Table 3** and **Figure 4**. Furthermore, we analyzed the number of effective FAVs carried by 17 F_2_ individuals ranging in height from 130 to 140 cm. As shown in **Figure 6B**, 11 out of 17 individual plants contained more than 50% of effective FAV markers, and one of the finest F_2_-98 individuals carried 20 effective FAV markers of FSH.

**Table 4:** Twenty effective favorable allelic variant SLAFs associated with flower stalk height trait and heterosis in the F_2_ population.

LG ID	SLAF ID	SNP Site	SNP in DH-17	SNP in DH-1	Proportion of heterozygote progeny in (90-100 cm]	Proportion of heterozygote progeny in (100-110 cm]	Proportion of heterozygote progeny in (110-120 cm]	Proportion of heterozygote progeny in (120-130 cm]	Proportion of heterozygote progeny in (130-140 cm]
LG2	Marker150989	103	G	A	0.27	0.33	0.42	0.47	0.47
LG2	Marker285135	157	C	T	0.27	0.33	0.38	0.44	0.35
LG2	Marker226058	73	T	A	0.27	0.25	0.39	0.53	0.53
LG8	Marker105154	31	C	A	0.36	0.37	0.43	0.43	0.65
LG8	Marker99568	114	G	A	0.27	0.39	0.46	0.47	0.53
LG8	Marker68370	68	A	G	0.27	0.39	0.42	0.47	0.65
LG8	Marker99657	175	G	T	0.27	0.35	0.46	0.46	0.59
LG8	Marker194036	54	A	G	0.18	0.29	0.43	0.41	0.47
LG8	Marker110725	107	G	A	0.27	0.33	0.41	0.44	0.65
LG8	Marker36724	137	T	C	0.27	0.39	0.47	0.56	0.59
LG8	Marker60226	101	A	G	0.27	0.39	0.45	0.44	0.47
LG8	Marker50587	44	A	G	0.27	0.37	0.42	0.52	0.65
LG8	Marker138614	81	T	C	0.18	0.37	0.43	0.49	0.65
LG8	Marker76340	119	T	C	0.18	0.35	0.46	0.43	0.53
LG8	Marker70349	90	T	C	0.27	0.39	0.43	0.54	0.65
LG8	Marker131660	47	A	G	0.27	0.39	0.42	0.48	0.59
LG8	Marker76408	112	A	T	0.27	0.41	0.44	0.51	0.65
LG8	Marker34305	27	A	G	0.27	0.41	0.47	0.52	0.65
LG8	Marker55369	114	G	A	0.36	0.41	0.49	0.54	0.65
LG8	Marker150308	172	C	A	0.18	0.35	0.38	0.48	0.53

**Table 5:** Detailed information of effective favorable allelic variant SLAF markers for the flower stalk height.

LG ID	SLAF Pos.	SLAF ID	SNP Num.	LOD	ADD	DOM	PVE%
LG2	59.822	Marker150989	1	5.55	2.80	2.98	7.19
LG2	64.263	Marker285135	2	5.17	2.83	2.39	6.43
LG2	64.263	Marker226058	4	5.17	2.83	2.39	6.43
LG8	66.010	Marker105154	2	6.42	2.91	1.75	6.07
LG8	66.010	Marker99568	3	6.42	2.91	1.75	6.07
LG8	66.010	Marker68370	1	6.42	2.91	1.75	6.07
LG8	66.010	Marker99657	2	6.42	2.91	1.75	6.07
LG8	66.010	Marker194036	3	6.42	2.91	1.75	6.07
LG8	66.157	Marker110725	1	6.38	2.90	1.77	6.08
LG8	66.304	Marker36724	2	6.48	3.00	1.60	6.28
LG8	66.304	Marker60226	1	6.48	3.00	1.60	6.28
LG8	66.304	Marker50587	2	6.48	3.00	1.60	6.28
LG8	66.304	Marker138614	2	6.48	3.00	1.60	6.28
LG8	66.304	Marker76340	2	6.48	3.00	1.60	6.28
LG8	66.304	Marker70349	5	6.48	3.00	1.60	6.28
LG8	66.304	Marker131660	5	6.48	3.00	1.60	6.28
LG8	66.304	Marker76408	1	6.48	3.00	1.60	6.28
LG8	66.304	Marker34305	2	6.48	3.00	1.60	6.28
LG8	66.304	Marker55369	3	6.48	3.00	1.60	6.28
LG8	66.598	Marker150308	3	5.40	2.63	1.85	5.17

## Discussion

### Construction of a high-resolution genetic map based on onion double haploid lines

To analyze an onion that is a cross-pollinated plant, with a large, repeated genome (Khosa et al., 2016; Finkers et al., 2021), DH lines are the most ideal first choice for mapping segregated populations. A sterile yellow doubled haploid line (DH-1) and a fertile yellow doubled haploid line (DH-17) were used to produce an F_2_ mapping population (containing 321 individuals) of onions in this study. The SLAF-seq strategy we applied was based on high-throughput sequencing technologies and developed as a simplified genome sequencing method (Sun et al., 2013). The advantages of using SLAF-seq technology rather than other available methods (such as RAD, GBS) is that SLAF adopted of a flexible double enzyme protocol and duplex barcode system, which could ensure the filtered molecular markers more uniformly distributed on the genome (Baird et al., 2008; Elshire et al., 2011; Sun et al., 2013), and this method uses high depth sequencing to define *de novo* genotypes without reference genome sequence and reference SNP, which is suitable for constructing onion genetic map.

In recent years, SLAF-seq has been widely applied in constructing high-resolution genetic maps not only for crops with reference genomes (Dai et al., 2018; Wang et al., 2018; Wei et al., 2020; Tao et al., 2022), but also for plants without a reference genome, such as orchardgrass (Zhao et al., 2016) and pea (Zheng et al., 2018). Detailed information on *Allium cepa* DHCU066619 genome has not yet been published, and its huge genome (14.9 Gb) consists of complex highly repetitive regions with (retro) transposons (Finkers et al., 2021). Therefore, we used the SLAF-seq technique to build a high-quality genetic map of onion without reference genome in 2018. To date, although several onion genetic maps have been constructed (Duangjit et al., 2013; Jo et al., 2017; Choi et al., 2020; Ryu et al., 2021), we used 10,584 polymorphic markers with the complete degree of 96.01% to construct onion genetic linkage map of 8 linkage groups (LGs) with a total length of 928.32 cM. The average marker distributed in the 8 LGs was 1,323, but only 198 markers were developed from LG6. Referred to the relevant literature on the onion genetic map and found that markers developed for each LG were about 50-200 (Choi et al., 2020; Cho et al., 2021; Fujito et al., 2021), and took into account large number of repetitive sequences existing in the onion genome (Finkers et al., 2021), we believed that the quality of this genetic map was not affected, although the number of markers located in the LG6 was low. As a whole, the linkage map carries the largest number of marker loci among all onion linkage maps constructed to date. Combined with the high depth of parental sequencing, low missing data rate information, average interval between markers (0.09 cM), and max gap value (**Table 2**; **Figure 3A**; **Supplementary Table S6**), the linkage map constructed in our study has the highest quality and resolution among onion linkage maps. Although total genetic map length was very small comparatively to the genome size of onion, our high-resolution genetic map should not only be useful for accurate and reliable mapping for QTLs, but also has important reference significance for onion DH-17 genome assembly. However, the chromosomal identity of LGs has not been known yet in this study, we would try to construct a physical chromosome assignment map based transcriptome unigene marker (Fujito et al., 2021) or carry out onion DH-17 genome assembly to resolve this issue in the future work.

### Implications for onion cytoplasmic male-fertility marker

Because onion is a biennial plant with a long breeding cycle, it is crucial to develop effective molecular markers for fertility identification that can be used during the practical breeding of onion. Cytoplasmic male-sterility (CMS), a maternally inherited trait characterized by the inability to produce viable pollen grains in higher plants, has been widely applied for the production of F_1_ hybrid seeds in agronomic crops such as onion, broccoli, cabbage, and radish (Havey, 2004; Bang et al., 2011). There are two main sources of CMS systems in onion: CMS-S and CMS-T. For the CMS-S system, male fertility is controlled by a single nuclear gene and a cytoplasmic factor (Jones and Clarke, 1943), and there are three loci involved in fertility restoration in the CMS-T system (Schweisguth, 1973). Therefore, the CMS-S was more widely used for onion hybrid cultivars because of its stability in various environments (Havey, 1995; Havey, 2000). To date, there has been a great deal of research performed on molecular markers for CMS-S fertility identification that are relevant for onion breeding (Sato, 1998; Engelke et al., 2003; Kim et al., 2009; Yang et al., 2013; Huo et al., 2015).

In our study, the fertility and sterility of pollen of the F_2_ population segregated in a 3:1 ratio. The segregation ratio for the *MsMs: Msms: msms* individuals that are marked by co-segregated markers DNF-566 and DNS-357 (Yang et al., 2013) was 1:2:1, which was consistent with a 3:1 Mendelian segregation ratio. Coincidentally, only one QTL locus (with higher LOD and PVE values, **Figure 3B**) related to male-fertile trait was detected on this genetic map, which may be the same *qtl* that was mapped by King et al. (1998). To summarize, we were able to reproduce the mapping of the onion male-fertility QTL in our F_2_ population, which indicated the higher quality of our constructed genetic linkage map, and thus, it can be used to efficiently locate novel QTLs for important traits of onion.

### Implications for onion QTL preliminary mapping of FSH

Suitable plant height is an important agronomic trait for elite hybrid seeds, especially for gramineal crops where the top grain yield organ (grain) is supported by a stem, such as sorghum, rice, and wheat (Zhou et al., 2012; Li et al., 2015; Jiang et al., 2017), and plays a vital role in the final yield formation. For onion, FSH directly affects lodging resistance and is closely related to seed yield and quality. In this study, double haploids DH-1 and DH-17 originated from commercial F_1_ hybrids, and their FSHs were lower than those of the original inducing hybrids by more than 30% (**Figure 1E**). The FSH of the F_1_ hybrids crossed with DH-1×DH-17 was restored to that of the original hybrids, and the heights of the hybrids were highly uniform and consistent. This phenomenon belongs in the category of heterosis. After taking into account the phenotypic analysis of FSH and four QTLs (located on LG2, LG5, LG8), only 26.60% of the phenotypic variation of the F_2_ population could be explained (**Table 3**). Consistent with this result, Baldwin et al. (2014) revealed genome regions on three chromosomes associated with bolting using a low-density linkage map. We inferred that the height of the onion flower stalk may be controlled by a heterosis effect between multiple dwarfing genes. Analysis of effective FAVs of onion FSH indicated that the genetic essence of heterosis may be the comprehensive effect of various genes containing a dominance effect, additive effect, epistatic effect, complementary and superdominance effect, and interaction effect that varied with various traits or genes.

Based on the high-resolution genetic map constructed by DH lines, we conducted QTL mapping for FSH. The implications are: (1) to propose the hypothesis of heterosis of FSH in onion; (2) to develop KASP markers for SNPs or genes relevant to FSH, which will lay a foundation for the construction of a molecular marker-assisted breeding system, and provide new insights into the developmental genetic mechanisms of FSH.

## Conclusion

In conclusion, our study provided a high- resolution genetic map based on double haploid lines with SLAF-seq in onion. We conducted QTL mapping of onion male fertile trait to verify that the genetic map had the higher-quality and could be efficient to locate novel QTLs for important traits of onion. We also conducted QTL mapping for the flower stalk height trait and 20 out of 49 SLAF markers (in 3/4 QTLs) were considered as effective FAV markers, which were suitable to develop KASP markers for SNPs or genes relevant to flower stalk height in the future. These findings would lay a foundation for the construction of molecular marker-assisted breeding system and provide new insights into the developmental genetic mechanisms of flower stalk height in onion.

## Data availability statement

The datasets presented in this study can be found in online repositories. The names of the repository/repositories and accession number(s) can be found in the article/**Supplementary Material**.

## Author contributions

YL and XW conceived and designed the research. YL performed the main data analysis, manuscript drafting and revision. XW, ZW, and YH performed the experiments. BL and YH constructed the population, collected phenotypes and SLAF-seq. ZW, YY, and YS carried out the field work. All authors contributed to the article and approved the submitted version.
