# Supplementary material for: Construction of a high-resolution genetic map and identification of single nucleotide polymorphism markers relevant to flower stalk height in onion
Source: Front Plant Sci. 2023 Jan 31;14:1100691. doi: 10.3389/fpls.2023.1100691 (PMC9928573; doi:10.3389/fpls.2023.1100691)
Supplement: Supplementary file 2 [file Table_2.docx]

**Table S1** **List of type and number of SLAF marker**

| **Type** | **Polymorphic SLAF** | **Non-Polymorphic SLAF** | **Repetitive SLAF** | **Total SLAF** |
| --- | --- | --- | --- | --- |
| Number | 155,067 | 535,802 | 1,807 | 692,676 |
| Percentage (%) | 22.39 | 77.35 | 0.26 | 100 |

**Table S2 Genotype distribution and classified statistic of polymorphic SLAF marker**

| **Segregating Pattern** | **Paternal genotype** | **Maternal genotype** | **Offspring genotype** | **Num. of SLAFs** | **Percentage (%)** |
| --- | --- | --- | --- | --- | --- |
| abxcd | ab | cd | ac, ad, bc, bd | 815 | 0.87 |
| efxeg | ef | eg | ee, ef, eg, fg | 1,331 | 1.42 |
| abxcc | ab | cc | ac, bc | 1,493 | 1.60 |
| ccxab | cc | ab | ac, bc | 1,027 | 1.10 |
| hkxhk | hk | hk | hh, hk, kk | 4,492 | 4.80 |
| lmxll | lm | ll | lm, ll | 4,367 | 4.67 |
| nnxnp | nn | np | nn, np | 2,248 | 2.40 |
| aaxbb | aa | bb | F_2_ (aa, ab, bb) | 77,766 | 83.14 |

**Table S3 Detailed information of SLAF markers related to the onion male-fertility trait**

| **LG ID** | **SLAF Pos.** | **SLAF ID** | **SNP Num.** | **LOD** | **ADD** | **DOM** | **PVE%** |
| --- | --- | --- | --- | --- | --- | --- | --- |
| 8 | 84.83 | Marker83987 | 0 | 289.23 | -0.49 | 0.49 | 113.13 |
| 8 | 84.83 | Marker69819 | 4 | 289.23 | -0.49 | 0.49 | 113.13 |
| 8 | 84.83 | Marker107031 | 1 | 289.23 | -0.49 | 0.49 | 113.13 |
| 8 | 84.98 | Marker196885 | 1 | 289.23 | -0.49 | 0.49 | 113.13 |
| 8 | 84.98 | Marker164689 | 2 | 289.23 | -0.49 | 0.49 | 113.13 |
| 8 | 84.98 | Marker92278 | 1 | 289.23 | -0.49 | 0.49 | 113.13 |
| 8 | 85.12 | Marker172809 | 2 | 289.23 | -0.49 | 0.49 | 113.13 |
| 8 | 85.12 | Marker150957 | 1 | 289.23 | -0.49 | 0.49 | 113.13 |
| 8 | 85.12 | Marker80322 | 1 | 289.23 | -0.49 | 0.49 | 113.13 |
| 8 | 85.42 | Marker175821 | 2 | 241.64 | -0.49 | 0.49 | 110.31 |
| 8 | 85.42 | Marker80019 | 3 | 241.64 | -0.49 | 0.49 | 110.31 |

**Table S4 Detailed information of SLAF markers within 4 QTLs for the onion flower stalk height**

| **LG ID** | **SLAF Pos.** | **SLAF ID** | **SNP Num.** | **LOD** | **ADD** | **DOM** | **Exp%** |
| --- | --- | --- | --- | --- | --- | --- | --- |
| LG2 | 59.82 | Marker311469 | 3 | 5.55 | 2.80 | 2.98 | 7.19 |
| LG2 | 59.82 | Marker70524 | 3 | 5.55 | 2.80 | 2.98 | 7.19 |
| LG2 | 59.82 | Marker150059 | 4 | 5.55 | 2.80 | 2.98 | 7.19 |
| LG2 | 59.82 | Marker111037 | 1 | 5.55 | 2.80 | 2.98 | 7.19 |
| LG2 | 59.82 | Marker150989 | 1 | 5.55 | 2.80 | 2.98 | 7.19 |
| LG2 | 64.11 | Marker172387 | 2 | 5.55 | 2.86 | 2.48 | 6.65 |
| LG2 | 64.11 | Marker85111 | 3 | 5.55 | 2.86 | 2.48 | 6.65 |
| LG2 | 64.11 | Marker84452 | 1 | 5.55 | 2.86 | 2.48 | 6.65 |
| LG2 | 64.11 | Marker49687 | 1 | 5.55 | 2.86 | 2.48 | 6.65 |
| LG2 | 64.11 | Marker123527 | 1 | 5.55 | 2.86 | 2.48 | 6.65 |
| LG2 | 64.26 | Marker79285 | 5 | 5.17 | 2.83 | 2.39 | 6.43 |
| LG2 | 64.26 | Marker153921 | 1 | 5.17 | 2.83 | 2.39 | 6.43 |
| LG2 | 64.26 | Marker285135 | 2 | 5.17 | 2.83 | 2.39 | 6.43 |
| LG2 | 64.26 | Marker163035 | 5 | 5.17 | 2.83 | 2.39 | 6.43 |
| LG2 | 64.26 | Marker226058 | 4 | 5.17 | 2.83 | 2.39 | 6.43 |
| LG5 | 42.16 | Marker9198 | 1 | 5.83 | -2.92 | 1.15 | 6.46 |
| LG5 | 42.16 | Marker78404 | 1 | 5.83 | -2.92 | 1.15 | 6.46 |
| LG5 | 42.16 | Marker17418 | 1 | 5.83 | -2.92 | 1.15 | 6.46 |
| LG5 | 42.16 | Marker72394 | 1 | 5.83 | -2.92 | 1.15 | 6.46 |
| LG5 | 42.16 | Marker16905 | 4 | 5.83 | -2.92 | 1.15 | 6.46 |
| LG5 | 42.16 | Marker195998 | 1 | 5.83 | -2.92 | 1.15 | 6.46 |
| LG5 | 42.30 | Marker93129 | 2 | 5.94 | -2.99 | 0.83 | 6.45 |
| LG5 | 42.30 | Marker49408 | 4 | 5.94 | -2.99 | 0.83 | 6.45 |
| LG5 | 42.30 | Marker96698 | 3 | 5.94 | -2.99 | 0.83 | 6.45 |
| LG5 | 42.60 | Marker64898 | 1 | 5.54 | -2.83 | 0.73 | 5.74 |
| LG5 | 42.89 | Marker73619 | 1 | 5.16 | -2.68 | 0.41 | 4.95 |
| LG5 | 42.89 | Marker55360 | 2 | 5.16 | -2.68 | 0.41 | 4.95 |
| LG5 | 42.89 | Marker68797 | 1 | 5.16 | -2.68 | 0.41 | 4.95 |
| LG5 | 42.89 | Marker165755 | 3 | 5.16 | -2.68 | 0.41 | 4.95 |
| LG5 | 42.89 | Marker160034 | 1 | 5.16 | -2.68 | 0.41 | 4.95 |
| LG5 | 42.89 | Marker11337 | 1 | 5.16 | -2.68 | 0.41 | 4.95 |
| LG5 | 42.89 | Marker63131 | 1 | 5.16 | -2.68 | 0.41 | 4.95 |
| LG8 | 66.01 | Marker105154 | 2 | 6.42 | 2.91 | 1.75 | 6.07 |
| LG8 | 66.01 | Marker99568 | 3 | 6.42 | 2.91 | 1.75 | 6.07 |
| LG8 | 66.01 | Marker68370 | 1 | 6.42 | 2.91 | 1.75 | 6.07 |
| LG8 | 66.01 | Marker99657 | 2 | 6.42 | 2.91 | 1.75 | 6.07 |
| LG8 | 66.01 | Marker194036 | 3 | 6.42 | 2.91 | 1.75 | 6.07 |
| LG8 | 66.15 | Marker110725 | 1 | 6.38 | 2.90 | 1.77 | 6.08 |
| LG8 | 66.30 | Marker36724 | 2 | 6.48 | 3.00 | 1.60 | 6.28 |
| LG8 | 66.30 | Marker60226 | 1 | 6.48 | 3.00 | 1.60 | 6.28 |
| LG8 | 66.30 | Marker50587 | 2 | 6.48 | 3.00 | 1.60 | 6.28 |
| LG8 | 66.30 | Marker138614 | 2 | 6.48 | 3.00 | 1.60 | 6.28 |
| LG8 | 66.30 | Marker76340 | 2 | 6.48 | 3.00 | 1.60 | 6.28 |
| LG8 | 66.30 | Marker70349 | 5 | 6.48 | 3.00 | 1.60 | 6.28 |
| LG8 | 66.30 | Marker131660 | 5 | 6.48 | 3.00 | 1.60 | 6.28 |
| LG8 | 66.30 | Marker76408 | 1 | 6.48 | 3.00 | 1.60 | 6.28 |
| LG8 | 66.30 | Marker34305 | 2 | 6.48 | 3.00 | 1.60 | 6.28 |
| LG8 | 66.30 | Marker55369 | 3 | 6.48 | 3.00 | 1.60 | 6.28 |
| LG8 | 66.59 | Marker150308 | 3 | 5.40 | 2.63 | 1.85 | 5.17 |

**Table S6** **The proportion of Singleton, Miss and transversion/transition about mapping markers**

| **LG ID** | **Singleton (%)** | **Miss (%)** | **Trv/Tri** |
| --- | --- | --- | --- |
| LG1 | 0.00 | 0.76 | 0.43 |
| LG2 | 0.00 | 0.49 | 0.50 |
| LG3 | 0.00 | 0.37 | 0.47 |
| LG4 | 0.00 | 0.41 | 0.49 |
| LG5 | 0.01 | 0.46 | 0.47 |
| LG6 | 0.04 | 1.12 | 0.52 |
| LG7 | 0.01 | 0.58 | 0.50 |
| LG8 | 0.00 | 0.34 | 0.49 |

**Table S5 The proportion of heterozygote progeny carrying FAV markers in F_2_ population**

| **SLAF ID** | **FSH in（90-100 cm】** | | | **FSH in（100-110 cm】** | | | **FSH in（110-120 cm】** | | | **FSH in（120-130 cm】** | | | **FSH in（130-140cm】** | | |
| --- | --- | --- | --- | --- | --- | --- | --- | --- | --- | --- | --- | --- | --- | --- | --- |
|  | Heterozygous  genotype  individuals | All  individuals | Proportion | Heterozygous  genotype  individuals | All  individuals | Proportion | Heterozygous  genotype  individuals | All  individuals | Proportion | Heterozygous  genotype  individuals | All  individuals | Proportion | Heterozygous  genotype  individuals | All  individuals | Proportion |
| Marker311469 | 6 | 11 | 0.5455 | 17 | 51 | 0.3333 | 54 | 125 | 0.4320 | 58 | 117 | 0.4957 | 8 | 17 | 0.4706 |
| Marker70524 | 7 | 11 | 0.6364 | 20 | 51 | 0.3922 | 62 | 125 | 0.4960 | 69 | 117 | 0.5897 | 9 | 17 | 0.5294 |
| Marker150059 | 4 | 11 | 0.3636 | 19 | 51 | 0.3725 | 47 | 125 | 0.3760 | 60 | 117 | 0.5128 | 6 | 17 | 0.3529 |
| Marker111037 | 6 | 11 | 0.5455 | 20 | 51 | 0.3922 | 62 | 125 | 0.4960 | 63 | 117 | 0.5385 | 7 | 17 | 0.4118 |
| Marker150989 | 3 | 11 | 0.2727 | 17 | 51 | 0.3333 | 53 | 125 | 0.4240 | 55 | 117 | 0.4701 | 8 | 17 | 0.4706 |
| Marker172387 | 6 | 11 | 0.5455 | 14 | 51 | 0.2745 | 44 | 125 | 0.3520 | 56 | 117 | 0.4786 | 6 | 17 | 0.3529 |
| Marker85111 | 7 | 11 | 0.6364 | 18 | 51 | 0.3529 | 44 | 125 | 0.3520 | 67 | 117 | 0.5726 | 8 | 17 | 0.4706 |
| Marker84452 | 7 | 11 | 0.6364 | 21 | 51 | 0.4118 | 64 | 125 | 0.5120 | 72 | 117 | 0.6154 | 9 | 17 | 0.5294 |
| Marker49687 | 7 | 11 | 0.6364 | 22 | 51 | 0.4314 | 68 | 125 | 0.5440 | 70 | 117 | 0.5983 | 10 | 17 | 0.5882 |
| Marker123527 | 6 | 11 | 0.5455 | 17 | 51 | 0.3333 | 59 | 125 | 0.4720 | 72 | 117 | 0.6154 | 9 | 17 | 0.5294 |
| Marker79285 | 6 | 11 | 0.5455 | 21 | 51 | 0.4118 | 64 | 125 | 0.5120 | 75 | 117 | 0.6410 | 9 | 17 | 0.5294 |
| Marker153921 | 7 | 11 | 0.6364 | 16 | 51 | 0.3137 | 50 | 125 | 0.4000 | 62 | 117 | 0.5299 | 7 | 17 | 0.4118 |
| Marker285135 | 3 | 11 | 0.2727 | 17 | 51 | 0.3333 | 47 | 125 | 0.3760 | 51 | 117 | 0.4359 | 6 | 17 | 0.3529 |
| Marker163035 | 5 | 11 | 0.4545 | 13 | 51 | 0.2549 | 52 | 125 | 0.4160 | 55 | 117 | 0.4701 | 7 | 17 | 0.4118 |
| Marker226058 | 3 | 11 | 0.2727 | 13 | 51 | 0.2549 | 49 | 125 | 0.3920 | 62 | 117 | 0.5299 | 9 | 17 | 0.5294 |
| Marker9198 | 2 | 11 | 0.1818 | 33 | 51 | 0.6471 | 69 | 125 | 0.5520 | 68 | 117 | 0.5812 | 8 | 17 | 0.4706 |
| Marker78404 | 2 | 11 | 0.1818 | 28 | 51 | 0.5490 | 62 | 125 | 0.4960 | 58 | 117 | 0.4957 | 7 | 17 | 0.4118 |
| Marker17418 | 3 | 11 | 0.2727 | 27 | 51 | 0.5294 | 67 | 125 | 0.5360 | 58 | 117 | 0.4957 | 10 | 17 | 0.5882 |
| Marker72394 | 2 | 11 | 0.1818 | 28 | 51 | 0.5490 | 65 | 125 | 0.5200 | 60 | 117 | 0.5128 | 9 | 17 | 0.5294 |
| Marker16905 | 2 | 11 | 0.1818 | 30 | 51 | 0.5882 | 68 | 125 | 0.5440 | 63 | 117 | 0.5385 | 8 | 17 | 0.4706 |
| Marker195998 | 2 | 11 | 0.1818 | 24 | 51 | 0.4706 | 50 | 125 | 0.4000 | 42 | 117 | 0.3590 | 6 | 17 | 0.3529 |
| Marker93129 | 4 | 11 | 0.3636 | 28 | 51 | 0.5490 | 55 | 125 | 0.4400 | 54 | 117 | 0.4615 | 8 | 17 | 0.4706 |
| Marker49408 | 3 | 11 | 0.2727 | 25 | 51 | 0.4902 | 61 | 125 | 0.4880 | 47 | 117 | 0.4017 | 7 | 17 | 0.4118 |
| Marker96698 | 2 | 11 | 0.1818 | 26 | 51 | 0.5098 | 57 | 125 | 0.4560 | 54 | 117 | 0.4615 | 8 | 17 | 0.4706 |
| Marker64898 | 2 | 11 | 0.1818 | 25 | 51 | 0.4902 | 51 | 125 | 0.4080 | 51 | 117 | 0.4359 | 7 | 17 | 0.4118 |
| Marker73619 | 3 | 11 | 0.2727 | 27 | 51 | 0.5294 | 57 | 125 | 0.4560 | 57 | 117 | 0.4872 | 8 | 17 | 0.4706 |
| Marker55360 | 3 | 11 | 0.2727 | 27 | 51 | 0.5294 | 64 | 125 | 0.5120 | 59 | 117 | 0.5043 | 8 | 17 | 0.4706 |
| Marker68797 | 3 | 11 | 0.2727 | 26 | 51 | 0.5098 | 59 | 125 | 0.4720 | 57 | 117 | 0.4872 | 7 | 17 | 0.4118 |
| Marker165755 | 2 | 11 | 0.1818 | 23 | 51 | 0.4510 | 48 | 125 | 0.3840 | 51 | 117 | 0.4359 | 7 | 17 | 0.4118 |
| Marker160034 | 2 | 11 | 0.1818 | 27 | 51 | 0.5294 | 60 | 125 | 0.4800 | 57 | 117 | 0.4872 | 8 | 17 | 0.4706 |
| Marker11337 | 3 | 11 | 0.2727 | 31 | 51 | 0.6078 | 69 | 125 | 0.5520 | 71 | 117 | 0.6068 | 10 | 17 | 0.5882 |
| Marker63131 | 2 | 11 | 0.1818 | 29 | 51 | 0.5686 | 64 | 125 | 0.5120 | 63 | 117 | 0.5385 | 8 | 17 | 0.4706 |
| Marker105154 | 4 | 11 | 0.3636 | 19 | 51 | 0.3725 | 54 | 125 | 0.4320 | 50 | 117 | 0.4274 | 11 | 17 | 0.6471 |
| Marker99568 | 3 | 11 | 0.2727 | 20 | 51 | 0.3922 | 58 | 125 | 0.4640 | 55 | 117 | 0.4701 | 9 | 17 | 0.5294 |
| Marker68370 | 3 | 11 | 0.2727 | 20 | 51 | 0.3922 | 53 | 125 | 0.4240 | 55 | 117 | 0.4701 | 11 | 17 | 0.6471 |
| Marker99657 | 3 | 11 | 0.2727 | 18 | 51 | 0.3529 | 57 | 125 | 0.4560 | 54 | 117 | 0.4615 | 10 | 17 | 0.5882 |
| Marker194036 | 2 | 11 | 0.1818 | 15 | 51 | 0.2941 | 54 | 125 | 0.4320 | 48 | 117 | 0.4103 | 8 | 17 | 0.4706 |
| Marker110725 | 3 | 11 | 0.2727 | 17 | 51 | 0.3333 | 51 | 125 | 0.4080 | 52 | 117 | 0.4444 | 11 | 17 | 0.6471 |
| Marker36724 | 3 | 11 | 0.2727 | 20 | 51 | 0.3922 | 59 | 125 | 0.4720 | 65 | 117 | 0.5556 | 10 | 17 | 0.5882 |
| Marker60226 | 3 | 11 | 0.2727 | 20 | 51 | 0.3922 | 56 | 125 | 0.4480 | 51 | 117 | 0.4359 | 8 | 17 | 0.4706 |
| Marker50587 | 3 | 11 | 0.2727 | 19 | 51 | 0.3725 | 53 | 125 | 0.4240 | 61 | 117 | 0.5214 | 11 | 17 | 0.6471 |
| Marker138614 | 2 | 11 | 0.1818 | 19 | 51 | 0.3725 | 54 | 125 | 0.4320 | 57 | 117 | 0.4872 | 11 | 17 | 0.6471 |
| Marker76340 | 2 | 11 | 0.1818 | 18 | 51 | 0.3529 | 57 | 125 | 0.4560 | 50 | 117 | 0.4274 | 9 | 17 | 0.5294 |
| Marker70349 | 3 | 11 | 0.2727 | 20 | 51 | 0.3922 | 54 | 125 | 0.4320 | 63 | 117 | 0.5385 | 11 | 17 | 0.6471 |
| Marker131660 | 3 | 11 | 0.2727 | 20 | 51 | 0.3922 | 52 | 125 | 0.4160 | 56 | 117 | 0.4786 | 10 | 17 | 0.5882 |
| Marker76408 | 3 | 11 | 0.2727 | 21 | 51 | 0.4118 | 55 | 125 | 0.4400 | 60 | 117 | 0.5128 | 11 | 17 | 0.6471 |
| Marker34305 | 3 | 11 | 0.2727 | 21 | 51 | 0.4118 | 59 | 125 | 0.4720 | 61 | 117 | 0.5214 | 11 | 17 | 0.6471 |
| Marker55369 | 4 | 11 | 0.3636 | 21 | 51 | 0.4118 | 61 | 125 | 0.4880 | 63 | 117 | 0.5385 | 11 | 17 | 0.6471 |
| Marker150308 | 2 | 11 | 0.1818 | 18 | 51 | 0.3529 | 48 | 125 | 0.3840 | 56 | 117 | 0.4786 | 9 | 17 | 0.5294 |
